# Systemic Light Chain Amyloidosis Mimicking Rheumatic Disorders

**DOI:** 10.1155/2016/7649510

**Published:** 2016-11-29

**Authors:** Rohit R. Rao, Wai Chung Yong, Mary Chester Wasko

**Affiliations:** ^1^Department of Hematology/Oncology, Allegheny Health Network, 320 East North Avenue, Pittsburgh, PA 15212, USA; ^2^Department of Internal Medicine, The Mary Imogene Bassett Hospital, One Atwell Road, Cooperstown, NY 13326, USA; ^3^Lupus Center of Excellence, Allegheny Health Network, 4800 Friendship Avenue, Suite 2600, North Tower, Pittsburgh, PA 15224, USA

## Abstract

Secondary amyloidosis can complicate chronic inflammatory autoimmune diseases. However, the clinical findings of primary amyloidosis may mimic those of primary rheumatologic disorders. We present the case of a 53-year-old woman who presented with dystrophic nail changes, dry eyes, bilateral carpal tunnel syndrome, Raynaud's phenomenon, and high titer positive nucleolar pattern antinuclear antibody. She was initially misdiagnosed as having Undifferentiated Connective Tissue Disease (UCTD). On further workup, she was eventually diagnosed with lambda light chain systemic amyloidosis by abdominal fat pad biopsy. Her symptoms completely resolved after autologous stem cell transplantation. With this case, we would like to highlight the similarities in the clinical features between light chain amyloidosis and rheumatological disorders. We would also like to emphasize the importance of the prompt recognition of the clinical features of amyloidosis which are crucial to triggering appropriate diagnostic procedures, since early diagnosis is a key to improving outcomes in this disease with an otherwise poor prognosis.

## 1. Introduction

Secondary amyloidosis can complicate chronic inflammatory autoimmune diseases such as rheumatoid arthritis (RA) and ankylosing spondylitis. However, the clinical findings of primary amyloidosis may mimic those of primary rheumatologic disorders. We present the case of a 53-year-old woman with a history of hypertension, bilateral carpal tunnel syndrome, and irritable bowel syndrome (IBS) who presented for evaluation of fingernail changes for the previous three years. The nails had vertical ridges and had cracked to the nail base. Occasionally, she also had mild pallor and bluish discoloration of her fingers on exposure to cold, suggestive of Raynaud's phenomenon. She had had diarrhea alternating with constipation and mild abdominal cramping for 3 years. Colonoscopy was done a year earlier and showed only diverticulosis of sigmoid colon; she was given a diagnosis of IBS by a gastroenterologist. Other symptoms included diffuse alopecia, dry eyes, fatigue, and numbness in both forearms and fingers bilaterally. She had had unilateral carpal tunnel release in the prior year; tissue was not sent for pathologic examination. Family history was significant for her mother having myelofibrosis and acute leukemia, but there was no family history of autoimmune disease. Her physical examination was normal except for vertical ridging of dystrophic nails of the first, second, and third digits of both hands, diffuse alopecia with no scalp rash, scaling or scarring, and numbness in her first, second, and third digits of both hands with positive Phalen and Tinel test. She had no skin tightening, rashes, or telangiectasias. Complete blood count and complete metabolic panel including serum calcium, urinalysis, erythrocyte sedimentation rate, and C-reactive protein were unremarkable except for total protein of 5.4 g/dL (normal: 6.1–7.9 g/dL). She had a normal serum iron, vitamin B12, folic acid, TSH, free T4, and chest X-ray. Serologic testing revealed an ANA of 1 : 1280 in a nucleolar pattern. Antibodies to SSA, SSB, Smith, RNP, centromere, Scl-70, and dsDNA were negative; C3 and C4 were normal. She was initially diagnosed with Undifferentiated Connective Tissue Disease (UCTD) by her rheumatologist. But, given her neuropathy, serum protein electrophoresis (SPEP) was sent and it showed an increase in *α*2-globulin fraction (16.7%; normal: 8.9–14.5%) and decrease in *γ*-globulin level and fraction (0.4 and 7.0%, resp.; normal: 0.6–1.9 g/dL; 9.8–24.4%) with an albumin-to-globulin ratio of 1.4. Serum immunofixation did not show any evidence of monoclonal gammopathy. Serum-free light chain assay revealed elevated free lambda light chains of 34.22 mg/dL (normal: 0.57–2.63 mg/dL) with low free kappa light chains of <0.29 mg/dL (normal: 0.33–1.94 mg/dL) and global decrement of IgG, IgA, and IgM. Urine protein electrophoresis and immunofixation were unremarkable.

**Figure 1:**
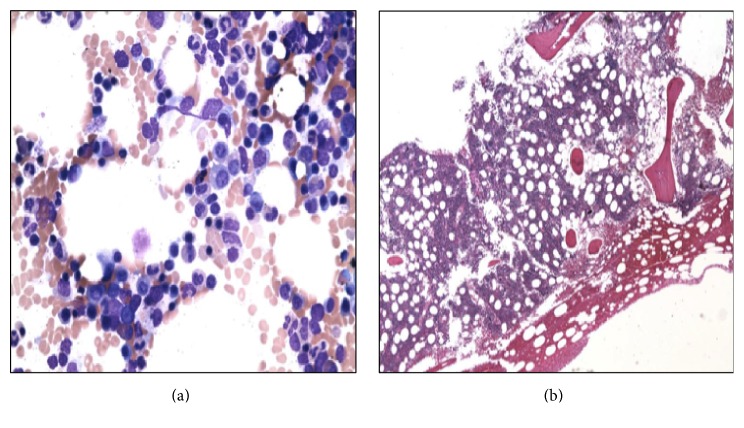
Bone marrow aspirate (a) and biopsy (b) showing scattered interstitial and focally clustered plasma cells and a small bland nonparatrabecular lymphoid aggregate.

**Figure 2:**
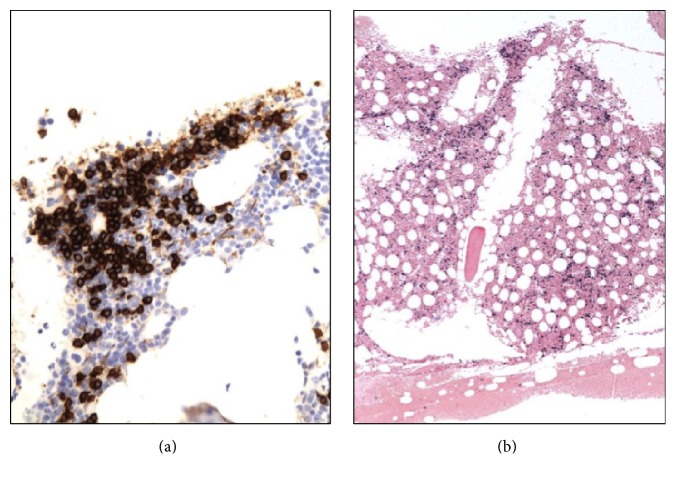
CD138 immunohistochemistry (a) highlighting interstitial and clustered plasma cells, estimated at 15% of total cells, with lambda light chain restriction by* in situ* hybridization (b).

**Figure 3:**
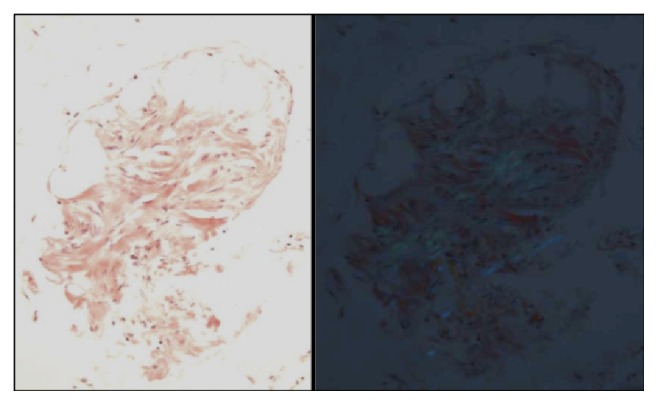
Abdominal fat pad biopsy showing positive Congo red staining.

She was referred to the Hematology-Oncology consultation team for further evaluation of the light chain monoclonal gammopathy. A bone marrow biopsy (Figures 1 and 2) showed normocellular bone marrow with 15% plasma cells and lambda light chain restriction; Congo red staining was negative. A skeletal survey, LDH, and *β*2-microglobulin were negative. Given her constellation of symptoms, amyloidosis remained a diagnostic concern. An abdominal fat pad biopsy was performed, revealing positive Congo red staining (Figure 3). Electron microscopic examination revealed clusters of filamentous material with average diameter of 8.7 nm, lined between lipid droplets, compatible with amyloid; no amyloid typing was performed. The patient was diagnosed with free lambda light chain amyloidosis. Echocardiogram, pro-BNP, and Troponin T were obtained for staging; these were within normal limits. The patient was treated with 4 cycles of bortezomib and dexamethasone. When near-complete remission was achieved with near-normalization of serum-free lambda light chains, decreasing to 3.07 mg/dL over 3 months, she subsequently underwent autologous peripheral blood stem cell transplantation after melphalan conditioning. Her posttransplantation serum-free lambda light chains decreased to 2.69 mg/dL at three months and then normalized to 2.57 mg/dL at six-month follow-up. The patient returned to her normal level of excellent function, with resolution of her initial symptoms, and returned to work one month after the transplant procedure. She had no clinical features of autoimmune disease. No posttransplant serologies were obtained.

## 2. Discussion

Deposits of amyloid may be distributed in many organs of the body (systemic amyloidosis) or may be restricted to a single organ (localized amyloidosis) [1, 2]. As demonstrated here, primary (AL) amyloidosis may mimic primary rheumatologic diseases such as SLE and Sjögren's syndrome. When this occurs, no typical pathologic findings of rheumatologic diseases are found in the biopsies of joints, salivary glands, or blood vessels except amyloid accumulation in these tissues [2, 3]. Others also have described amyloidosis in rheumatic diseases such as Sjögren's syndrome, scleroderma, and primary biliary cirrhosis as a secondary phenomenon [3, 4]. In our patient, dry eyes can likely be explained by the amyloid deposition in lacrimal glands, masquerading as Sjögren's syndrome [2]. AL amyloidosis associated with multiple myeloma may result in an arthropathy resembling RA [5]. In this setting, treating the underlying hematologic malignancy is the most important intervention as patients tend to respond better to multiple myeloma treatment than to treatment of the amyloid arthropathy. Sensitivity of selected physical and laboratory findings for detecting amyloidosis in cases of multiple myeloma (MM) associated arthropathy has been illustrated in literature (Table 1) [5]. While our patient did not have arthropathy, she had bilateral carpal tunnel syndrome and hypogammaglobulinemia, which raised the suspicion of AL amyloidosis. The carpal tunnel symptoms of the prior year are consistent with the typical 2-year delay between the onset of symptoms and the recognition of amyloidosis [6].

Amyloid-associated rheumatic disease manifestations in primary amyloidosis have been associated with an anticentromere antibody pattern of ANA [6]. However, to the best of our knowledge, a nucleolar pattern ANA has not been previously described in this setting. Given the association of a nucleolar pattern ANA and scleroderma, our patient warrants follow-up for the progression to the development of scleroderma, though this seems unlikely in the setting of a successful stem cell transplantation and resolution of symptoms.

Alopecia or nail dystrophy can rarely present as a sign of occult amyloidosis [7, 8]. Unusual manifestations of primary cutaneous amyloidosis in association with Raynaud's phenomenon and livedo reticularis have been reported [1], but, in this case, Raynaud's phenomenon preceded the cutaneous amyloidosis by ten years. Histopathology described in scalp biopsies of amyloid patients with alopecia has demonstrated that amyloid deposits were seen to be compressing pilosebaceous units, accompanied by atrophy of these structures and loss of hair from the shafts [9]. Nail biopsy may show amyloid deposits in papillary dermis of the matrix, nail bed, and perivascular areas, which perhaps cause vascular impairment with subsequent nail brittleness, crumbling, onycholysis, subungual thickening, striation, or anonychia [10].

Initially, the bone marrow biopsy in our patient was performed for the purpose of diagnosing plasma cell dyscrasia. The biopsy revealed lambda light chain restriction on* in situ* hybridization but negative Congo red staining. The diagnostic yield of bone marrow biopsy in primary amyloidosis is estimated at 63% [11, 12]. We interpreted that the false-negative results could be due to variable and sometimes pale staining of amyloid fibrils, inadequate material, or improper use of polarizing instruments. In addition, the correct interpretation of Congo red staining requires experience and is somewhat subjective [12]. Thioflavin staining is another option to confirm the validity of negative Congo red staining because it can detect smaller deposits, although it is less specific [12].

Abdominal fat pad aspiration has replaced most of the previously used tissue biopsies because it is easy to perform and safe and involves minimal patient discomfort and morbidity. Studies have shown that, in about 11 percent of cases, fat pad biopsy can be positive in the setting of negative bone marrow staining [6]. The sensitivity and specificity for abdominal fat pad aspiration are 55–85% and 75–100%, respectively [11, 12]. Labial salivary gland (LSG) biopsy is a good diagnostic modality to consider for diagnosing amyloidosis mimicking Sjögren's syndrome because it shares the advantages of abdominal fat pad aspiration with similar diagnostic yield and offers the ability to rule out Sjögren's syndrome simultaneously. The diagnostic yield of LSG is roughly 58–86%, with specificity of 100% [13, 14]. Biopsy of another involved organ such as kidney, liver, colon, or heart can be utilized as well due to the high sensitivity (87–98%), if Congo red staining on the bone marrow and abdominal fat pad biopsy is negative, but there is high clinical suspicion of amyloidosis [11, 12].

To summarize, our patient presented with dystrophic nail changes, carpal tunnel syndrome, Raynaud's phenomenon, and high titer positive nucleolar pattern ANA and was diagnosed with lambda light chain systemic amyloidosis by abdominal fat pad biopsy. Fortunately, in our case, the diagnosis was made before the development of cardiomyopathy, a complication significantly reducing life expectation [15]. Based on the Revised Mayo Clinic Staging for amyloidosis [15], our patient was staged as Stage II, which has median survival of 97 months in stem cell transplant eligible patients versus 19 months in ineligible patients. Thus we treat amyloidosis early on, since prognosis in untreated AL amyloidosis is dismal. We suggest further monitoring for her positive nucleolar pattern ANA due to the possible progression to scleroderma or other rheumatic diseases, though this would be a remote possibility following her bone marrow transplantation. Furthermore, due to the relatively low diagnostic yield of bone marrow biopsy, we employed an alternate diagnostic modality with little morbidity (abdominal fat pad aspiration) to establish this patient's diagnosis. Our case also highlights the many similarities in the clinical features between systemic light chain amyloidosis and primary rheumatological disorders. It also underlines the importance of the prompt recognition of “red flags” of amyloidosis which are crucial to triggering appropriate diagnostic procedures, since early diagnosis prior to the development of advanced cardiomyopathy is a key to improving outcomes in this disease.

## Figures and Tables

**Table 1:** Sensitivity of selected physical and laboratory findings for detecting amyloidosis in the setting of multiple myeloma [5].

Finding	Sensitivity (%)
(1) Macroglossia, carpal tunnel syndrome, or shoulder pads (any one)	47
(2) Hypercalcemia	26
(3) Hypogammaglobulinemia	22
(4) Renal failure	23
(5) Juxta-articular bone lesions	25
(6) Bence Jones proteinemia	56
(1), (2), (3), or (4)	67
(1), (2), (3), (4), or (5)	74
(1), (2), (3), (4), or (6)	86
(1), (2), (3), (4), (5), or (6)	91

## References

[1] Naldi L., Marchesi L., Locati F., Berti E., Cainelli T. (1992). Unusual manifestations of primary cutaneous amyloidosis in association with Raynaud's phenomenon and livedo reticularis. *Clinical and Experimental Dermatology*.

[2] Duna G. F., Cash J. M. (1996). Rheumatic manifestations of dysproteinemias and lymphoproliferative disorders. *Rheumatic Disease Clinics of North America*.

[3] Breathnach S. M. (1988). Amyloid and amyloidosis. *Journal of the American Academy of Dermatology*.

[4] Azon-Masoliver A. (1995). Widespread primary localized cutaneous amyloidosis (macular form) associated with systemic sclerosis. *British Journal of Dermatology*.

[5] Elsaman A. M., Radwan A. R., Akmatov M. K. (2013). Amyloid arthropathy associated with multiple myeloma: a systematic analysis of 101 reported cases. *Seminars in Arthritis and Rheumatism*.

[6] Gertz M. A., Merlini G., Treon S. P. (2004). Amyloidosis and Waldenström's macroglobulinemia. *Hematology. American Society of Hematology, Education Program*.

[7] Fanti P. A., Tosti A., Morelli R., Galbiati G. (1991). Nail changes as the first sign of systemic amyloidosis. *Dermatologica*.

[8] Hunt S. J., Caserio R. J., Abell E. (1991). Primary systemic amyloidosis causing diffuse alopecia by telogen arrest. *Archives of Dermatology*.

[9] Brownstein M. H., Helwig E. B. (1970). The cutaneous amyloidosis. II. Systemic forms. *Archives of Dermatology*.

[10] Prat C., Moreno A., Viñas M., Jucglà A. (2008). Nail dystrophy in primary systemic amyloidosis. *Journal of the European Academy of Dermatology and Venereology*.

[11] Bowen K., Shah N., Lewin M. (2012). AL-amyloidosis presenting with negative congo red staining in the setting of high clinical suspicion: a case report. *Case Reports in Nephrology*.

[12] Hachulla E., Grateau G. (2002). Diagnostic tools for amyloidosis. *Joint Bone Spine*.

[13] Hachulla E., Janin A., Flipo R. M. (1993). Labial salivary gland biopsy is a reliable test for the diagnosis of primary and secondary amyloidosis. A prospective clinical and immunohistologic study in 59 patients. *Arthritis and Rheumatism*.

[14] Foli A., Palladini G., Caporali R. (2011). The role of minor salivary gland biopsy in the diagnosis of systemic amyloidosis: results of a prospective study in 62 patients. *Amyloid*.

[15] Kumar S., Dispenzieri A., Lacy M. Q. (2012). Revised prognostic staging system for light chain amyloidosis incorporating cardiac biomarkers and serum free light chain measurements. *Journal of Clinical Oncology*.

